# Molecular Dynamics Simulations Predict that rSNP Located in the *HNF-1α* Gene Promotor Region Linked with MODY3 and Hepatocellular Carcinoma Promotes Stronger Binding of the HNF-4α Transcription Factor

**DOI:** 10.3390/biom10121700

**Published:** 2020-12-21

**Authors:** Eva Španinger, Uroš Potočnik, Urban Bren

**Affiliations:** 1 Faculty of Chemistry and Chemical Engineering, University of Maribor, Smetanova ulica 17, SI-2000 Maribor, Slovenia; eva.spaninger@gmail.com (E.Š.); uros.potocnik@um.si (U.P.); 2 Faculty of Medicine, University of Maribor, Taborska 8, SI-2000 Maribor, Slovenia; 3 Faculty of Mathematics, Natural Sciences and Information Technologies, University of Primorska, Glagoljaška 8, SI-6000 Koper, Slovenia

**Keywords:** HNF-1α, HNF-4α, MODY3, rSNP, molecular dynamics, binding free energy

## Abstract

Our study aims to investigate the impact of the Maturity-onset diabetes of the young 3 disease-linked rSNP rs35126805 located in the *HNF-1α* gene promotor on the binding of the transcription factor HNF-4α and consequently on the regulation of *HNF-1α* gene expression. Our focus is to calculate the change in the binding affinity of the transcription factor HNF-4α to the DNA, caused by the regulatory single nucleotide polymorphism (rSNP) through molecular dynamics simulations and thermodynamic analysis of acquired results. Both root-mean-square difference (RMSD) and the relative binding free energy ΔΔ*G_bind_* reveal that the HNF-4α binds slightly more strongly to the DNA containing the mutation (rSNP) making the complex more stable/rigid, and thereby influencing the expression of the *HNF-1α* gene. The resulting disruption of the HNF-4α/HNF-1α pathway is also linked to hepatocellular carcinoma metastasis and enhanced apoptosis in pancreatic cancer cells. To the best of our knowledge, this represents the first study where thermodynamic analysis of the results obtained from molecular dynamics simulations is performed to uncover the influence of rSNP on the protein binding to DNA. Therefore, our approach can be generally applied for studying the impact of regulatory single nucleotide polymorphisms on the binding of transcription factors to the DNA.

## 1. Introduction

Studying the effect of single nucleotide polymorphisms (SNPs) in the coding part of the genome directly impacting protein structures has been at the center of attention for the vast majority of the scientific community. On the other hand, besides the somatic and germline mutations in oncogenes and tumor-suppressor genes, genome-wide association studies (GWASes) suggest that even germline single-nucleotide polymorphisms located in introns are associated with altered cancer risks [1]. The poorly investigated non-coding part of the genome also contains sequences for the regulation of gene expression [2,3]. Consequentially, only a few available databases on regulatory SNPs (rSNPs) exist, and their biological roles in disease (cancer) development, progression, and response to therapy ought to be investigated further [1,4].

Even though the Protein Data Bank (PDB) contains an ever-increasing number of structures, finding a human transcription factor-DNA complex especially highly homologous to a sequence containing rSNPs represents quite a challenge. Therefore, we had only a few options to choose from. We have successfully found a complex of hepatocyte nuclear factor 4 alpha (HNF-4α) with DNA. A study of an Italian family linked the SNP in the *hepatocyte nuclear factor 1 alpha (HNF-1α)* gene regulatory region with the Maturity-onset diabetes of the young 3 (MODY3) as a result of disruption of the transcription factor HNF-4α binding site [5].

Accounting for up to two percent of all diabetes cases in the United States, MODY represents the prevailing form of monogenic diabetes [6,7]. Various gene mutations demonstrated to cause MODY decrease the ability of the pancreas to produce insulin, resulting in high blood glucose levels and, with time, even in damaged body tissues, particularly the eyes, kidneys, nerves, and blood vessels [7]. Based on the gene mutations, MODY can be divided into six types among, which MODY3 represents the most frequent one. MODY3 is linked with an autosomal dominant mutation in the Transcription Factor 1 gene that affects the HNF-1α protein [5,7]. Even though MODY3 represents a permanent type of diabetes, it can be, with a few exceptions, initially treated using a special diet and/or oral sulfonylureas [5,7].

The hepatocyte nuclear factors (HNFs) are already regarded as promising biomarkers for prognostic predictions and as potential therapeutic targets in cancer since they play an important role in the sustenance of solid tumors, however with numerous associated downstream targets they are not easily druggable [8,9]. The *HNF-1α* gene encodes for homeobox A protein nuclear transcription factor essential for the expression of several hepatic genes participating in detoxification, homeostasis, and metabolism of glucose, lipids, steroids, and amino acids [5,10]. Reported consequences of mutations in this gene include MODY3, type II diabetes, hepatic adenomas, and pancreatic ductal adenocarcinoma [7,10,11,12,13]. The expression of the *HNF-1α* gene is regulated by the transcription factor HNF-4α that binds DNA as a homodimer [8,14]. It is presumed that this gene plays a role in the development of the liver, kidneys, and intestines [14]. It has been found that both *HNF-1α* and *HNF-4α* regulate not only the function of differentiated beta-cells, but the growth and function of islet beta-cells as well [11]. Irregular expression or mutations in the *HNF-4α* gene promote or prevent definitive endoderm differentiation, cause monogenic autosomal dominant non-insulin-dependent diabetes mellitus type I, and possibly contribute to the progression of hepatocellular carcinoma [14,15,16]. Additionally, the disruption of the HNF-4α/HNF-1α pathway may form the important molecular mechanism of renal cell carcinogenesis [8].

Molecular dynamics (MD) simulations have been previously used for exploring the DNA binding sites of various proteins (including transcription factors) exploiting three different approaches. In the first one, the focus is on the DNA and its behavior to reveal the recognition/binding motifs on the DNA [17] and/or to improve the accuracy in the prediction of binding sites [18]. The second one focuses on the protein structure and how various mutations in proteins or their specific domains affect their ability to bind to the DNA [19,20,21,22]. The third approach focuses on studying the protein–DNA complexes to uncover types of interactions between them and the binding affinity of proteins towards DNA [23,24,25,26].

A majority of studies focus on large variations in DNA or protein structures, however, even the smallest variation like single nucleotide polymorphism (SNP) can have detrimental [27] and even oncogenic effects [1]. 

The aim of our study is to elucidate the impact of the MODY3 disease-linked rSNP rs35126805 located in the non-coding DNA region, in the *HNF-1α* gene promotor -58 (upstream), on the binding of the transcription factor HNF-4α, and the regulation of *HNF-1α* gene expression. Our focus was to investigate the change in the binding affinity of the transcription factor HNF-4α to the DNA, caused by the studied rSNP through molecular dynamics simulations and thermodynamic analysis of acquired results. To our knowledge, this is the first study where thermodynamic analyses of the obtained results from molecular dynamics simulations were performed to uncover the influence of rSNP on the protein binding to DNA.

Both atom-positional root-mean-square difference (RMSD) and the binding free energy Δ*G_bind_* reveal that there is a significant difference between the structures and behavior of the DNA-HNF-4α complex including the wild-type base pair and the rSNP. Therefore, our approach can be generalized for studying the impact of regulatory single nucleotide polymorphisms on the binding of transcription factors to the DNA.

## 2. Methods

### 2.1. Preparation of the Starting Structures for the MD Simulation

The complex of transcription factor HNF-4α with DNA was obtained from the Protein Data Bank (PDB) using the entry code 3CBB. For finding and exploring the DNA sequence and its location on the human genome, the ENSEMBL genome browser [28] and its tool BLAST [28] were employed. The ENSEMBL additionally facilitated the uncovering of the location of rSNP in the *HNF-1α* gene promotor -58 (upstream). Even though in the ENSEMBL and dbSNP [29] our studied rSNP labeled rs35126805 has no reported (severe) clinical significance, it evidentially causes MODY3 as a consequence of disruption of the HNF4-α transcription factor binding site, thereby effecting the *HNF-1α* gene transcription [5,6,7].

A part of the DNA HNF-4α complex with PDB entry code 3CBB represents Zn ions bound to four cysteine amino acid residues, andthus opening a new challenge on how to represent the coordinative bonds, meaning how to distribute the partial charges between the Zn ion and the cysteines (Cys) for subsequent molecular dynamics simulations. The idea of including the parameters for Zn ions in simulations by Marco and coworkers [18] seemed to be a relatively accurate solution for representing the coordinative bonds in our complex. By using the parameters found in the recent versions of AMBER force field parameter files, we complemented the parameter files and created new definitions for Zn ions and their coordinating Cys residues in the libraries of the Molecular Dynamics Package Q version 5.0 [30]. The parameters for Zn ions were obtained from the AMBER force field parameter file amber99.par designed by Hoops, Anderson, and Merz [31]. The AMBER force field parameters for the sulfur ions of cysteine residues coordinatively bound to zinc ions were developed by Peters and coworkers [32].

In the Crystallographic Object-Oriented Toolkit (Coot) program [33], we induced a mutation on the wild-type adenine–thymine base pair and replaced it with the rSNP cytosine–guanine base pair. To prevent protein unfolding, acetyl groups and methionine groups were added to C-terminal and N-terminal amino-acid residues, respectively, in the PyMOL Molecular Graphics System [34]. Certain amino-acid residues and ultimate DNA base pairs (3’ and 5’) were renamed to match the defined ones in the Q libraries.

Using the AMBER force field implemented in the Molecular Dynamics Package Q [30], the configurational ensembles for the evaluation of free energies were generated from molecular dynamics trajectories. In simulations, our solute molecules were immersed in a sphere (30 Å radius) of TIP3P water molecules subjected to the surface-constraint, all-atom solvent (SCAAS)-type boundary conditions [35], as implemented in the Molecular Dynamics Package Q [30]. The imposed constraints are designed to mimic an infinite aqueous solution. Therefore, stable and realistic free energies are produced even in the small spherical models (water droplets) compared to large simulation boxes utilizing periodic boundary conditions [36,37]. Testing the proper water sphere size between 23 and 30 Å, as well as finding the best position for its center using short molecular dynamics simulations with the Qprep program of Molecular Dynamics Package Q [30] on the CROW computer cluster located on The National Institute of Chemistry in Ljubljana, has revealed poor stability of the dangling DNA end. Therefore, we decided to elongate the dangling part of the DNA. Its sequence was obtained from the ENSEMBL genome browser [28], and the x3DNA program v.2.3 [38] was used for its build-up. Several translations and rotations in the PyMOL Molecular Graphics System [34] were needed to get a proper fitting of the newly built DNA segment to the prepared complex. The newly built DNA and the prepared complex were then joined. To optimize the contacts between the original and additionally built DNA, a short molecular dynamics simulation with the Qprep program (Q-Amber 95 force field) was performed. The charges of ionized amino-acid residues and DNA base pairs close to the solvent-sphere boundary were reduced. [39] Finally, sodium ions were added to neutralize the system. However, just neutralizing the system might lead to certain artefacts, as the dynamics of DNA strongly depend on the ion concentrations [40].

The reference double-stranded DNA systems were obtained by removing the HNF-4α portion of the complex and by adjusting the number of sodium counterions.

### 2.2. Molecular Dynamics Simulations

For a proper analysis of the influence of rSNP in DNA on the HNF-4α binding, we prepared four separate systems for subsequent molecular dynamic simulations. The first system was the elongated wild-type DNA, the second was the elongated mutated DNA with rSNP. The third and fourth systems were DNA-HNF-4α complex with wild-type and rSNP base pair, respectively, and of course with all the preparations described in the previous paragraph. Preparatory simulations in the Qprep program with 30 Å radius water solvation sphere for obtaining topology files were performed on all four systems and bond lengths, bond angles, and torsions as well as improper angles were checked. As sodium counterions were placed randomly in the water sphere, replacing some of the solvent (water) molecules, we had to change some of their positions as they were too close to the sphere’s outside boundary or too close to the DNA or the protein part of the complex. Next, we performed four series of 2 ns long equilibrating MD simulations with the Qdyn program on all four systems to obtain the initial velocities and to slowly heat-up the system to the production simulation temperature of 298.15 K as well as to loosen heavy-atom restraints that prevent the explosion of the system during equilibration. On the equilibrated systems, sixteen 5 ns long unrestrained production runs were performed using the Qdyn program. A 2 fs step size was used along with the shake algorithm for bonds involving hydrogen atoms [41].

DNA and protein atoms protruding beyond the sphere boundaries were restrained to their initial coordinates using harmonic restraints. Nonbonding interactions of these atoms were turned off. Nonbonding interactions between the atoms inside the simulation sphere were subjected to a 10 Å cut-off. The local-reaction field method was used to treat long-range electrostatic interactions for distances beyond this cut-off. [36,42] Energies were sampled every 10 steps.

### 2.3. Analysis

We aimed to uncover the influence of rSNP on the binding of transcription factor HNF-4α to the DNA in the *HNF-1α* gene promotor. The analysis was performed in the Visual Molecular Dynamics (VMD) program [43]. The atom-positional root-mean-square difference (RMSD) was calculated for all systems yielding structural differences between conformations.

#### 2.3.1. Analysis of Intermolecular Interactions

Hydrogen bonds exert a great influence on the binding, especially when dealing with nucleic acids and proteins solvated in the water where they represent intra- and intermolecular interactions. Therefore, we examined their quantity, frequency of occurrence, and duration. The water-mediated hydrogen bonds between the protein and the DNA in the 10 Å radius around the rSNP were also considered. The hydrogen-bond existence criteria were strictly geometrical: The donor–acceptor distance was shorter than 3.0 Å and hydrogen–donor–acceptor angle was smaller than 20°.

#### 2.3.2. Thermodynamic Analysis

For the analysis of the binding affinity, the Linear Interaction Energy (LIE) method was used, where the binding free energy is calculated based on the average interaction energies. The LIE method involves molecular dynamics simulations of only two physical states—the bonded and the free ligand. The main idea is to separately treat the contributions of the electrostatic and van der Waals interactions to the total binding affinity. According to the LIE method, the binding free energy Δ*G_bind_* is calculated using the following equation:

(1) $$\Delta G_{bind}=\alpha \times \left(\langle E_{vdW}^{l-s}\rangle {}_{bound}-\langle E_{vdW}^{l-s}\rangle {}_{unbound}\right)+\beta \times \left(\langle E_{el}^{l-s}\rangle {}_{bound}-\langle E_{el}^{l-s}\rangle {}_{unbound}\right)$$

$\Delta G_{bind}$ the binding free energy (kcal/mol);
$\langle E_{vdW}^{l-s}\rangle$ average vdW interaction energies between the ligand and it’s surrounding (kcal/mol);
$\langle E_{el}^{l-s}\rangle$ average electrostatic interaction energies between the ligand and it’s surrounding (kcal/mol);
*l* ligand;
*s* surrounding environment;
*α, β* empirical parameters of the LIE method.

The two above-mentioned physical states encompass the free ligand (in our case the DNA in the water solvation sphere) and the bonded ligand (in our case the complex of HNF-4α bound to the DNA in the water solvation sphere).

Series of both the van der Waals and the electrostatic interaction energies were obtained from the energy trajectories with the Qfep program. From the average interaction energies, the binding free energy Δ*G_bind_* was calculated using Equation (1). The following preoptimized values were applied for the LIE empirical parameters α = 0.18 and β = 0.43 [44,45].

**Scheme 1:**
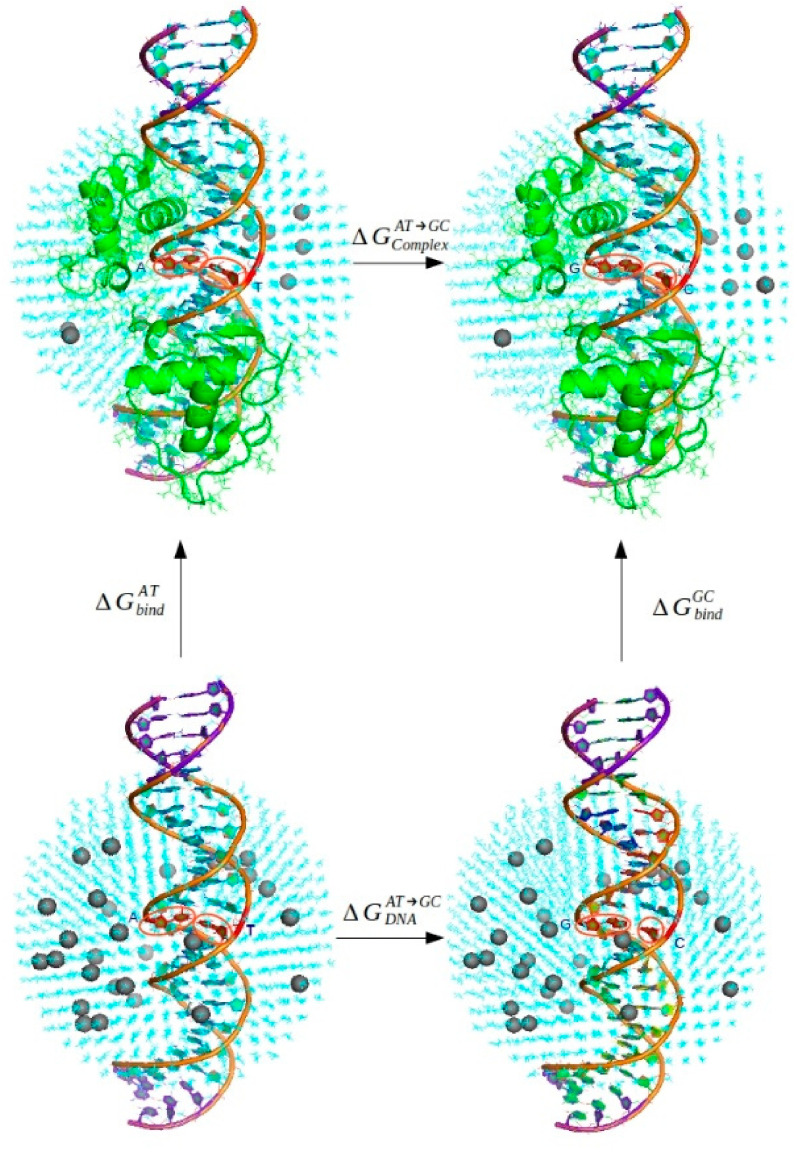
The four investigated systems in solvated water spheres prepared for the subsequent molecular dynamics simulations: Upper left—wild-type complex; upper right—mutated rSNP rs35126805 containing complex; lower left—wild-type DNA, mutated rSNP rs35126805 containing DNA.

To obtain the difference between the binding free energies of the wild-type complex and the mutated rSNP rs35126805 containing complex, Equation (2) was derived based on Scheme 1 and Equation (1).

(2) $$\begin{array}{c} \Delta G_{bind}^{AT}+\;\Delta G_{Complex}^{AT\to GC}-\;\Delta G_{bind}^{GC}-\;\Delta G_{DNA}^{AT\to GC}=\;0\\ \Delta \Delta G_{bind}=\;\Delta G_{bind}^{GC}-\;\Delta G_{bind}^{AT}=\Delta G_{Complex}^{AT\to GC}-\Delta G_{DNA}^{AT\to GC}\;\\ \Delta \Delta G_{bind}=\alpha \times \left(\langle E_{vdW}^{GC-s}\rangle {}_{Complex}-\langle E_{vdW}^{GC-s}\rangle {}_{DNA}\right)+\beta \times \left(\langle E_{el}^{GC-s}\rangle {}_{Complex}-\langle E_{el}^{GC-s}\rangle {}_{DNA}\right)\\ -\alpha \times \left(\langle E_{vdW}^{AT-s}\rangle {}_{Complex}-\langle E_{vdW}^{AT-s}\rangle {}_{DNA}\right)\\ -\beta \times \left(\langle E_{el}^{AT-s}\rangle {}_{Complex}-\langle E_{el}^{AT-s}\rangle {}_{DNA}\right) \end{array}$$

$\Delta G_{bind}$ the binding free energy (kcal/mol);
$\Delta \Delta G_{bind}$ the difference between the binding free energy of the wild-type complex and the mutated rSNP rs35126805 containing complex (kcal/mol);
$\langle E_{vdW}^{l-s}\rangle$ average van der Waals interaction energies between the ligand and it’s surrounding (kcal/mol);
$\langle E_{el}^{l-s}\rangle$ average electrostatic interaction energies between the ligand and it’s surrounding (kcal/mol);
*l* ligand: AT—wild-type base pair, GC—mutated rSNP base pair; Complex—complex of HNF-4α transcription factor bound to the DNA, DNA—solely the DNA double helix;
*s* surrounding environment included in the water solvent sphere;
*α, β* empirical parameters of the LIE method.

The Linear Interaction Energy (LIE) method addresses the entropic terms implicitly by parametrizing the values of its empirical parameters α and β on a large set of experimental binding free energies and by performing molecular dynamics simulations on both end-states. It was observed before that the absolute binding free energies of LIE predictions are not particularly reliable, but that the relative (ΔΔ*G_bind_*) ranking of binding free energies can still be useful, as it usually correlates with the relative rankings in experiments [46,47].

## 3. Results and Discussion

As our goal was to uncover the influence of rSNP on the binding of transcription factor (TF) HNF-4α to the *HNF-1α* gene promotor DNA sequence, we focused on elucidating the differences between HNF-4α bound to the DNA containing SNP and to the DNA containing the wild-type base pair.

**Figure 1:**
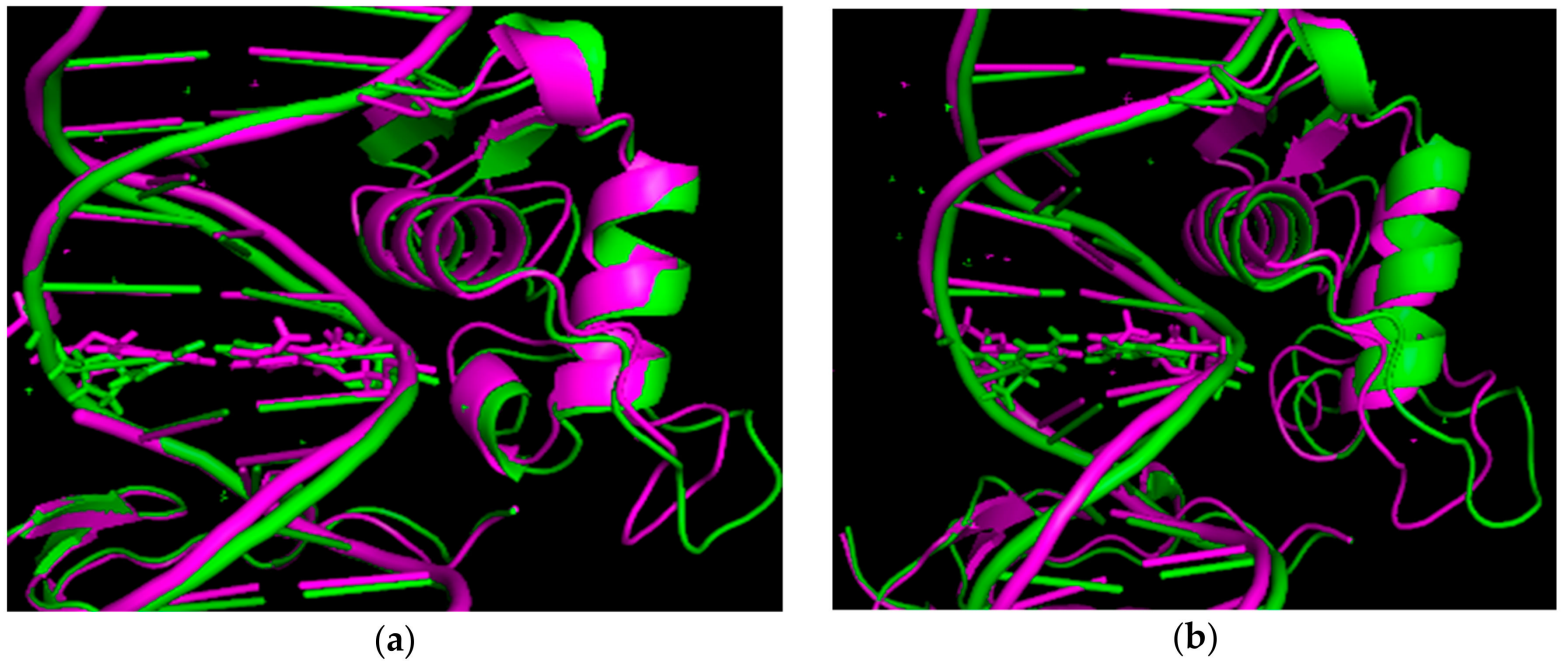

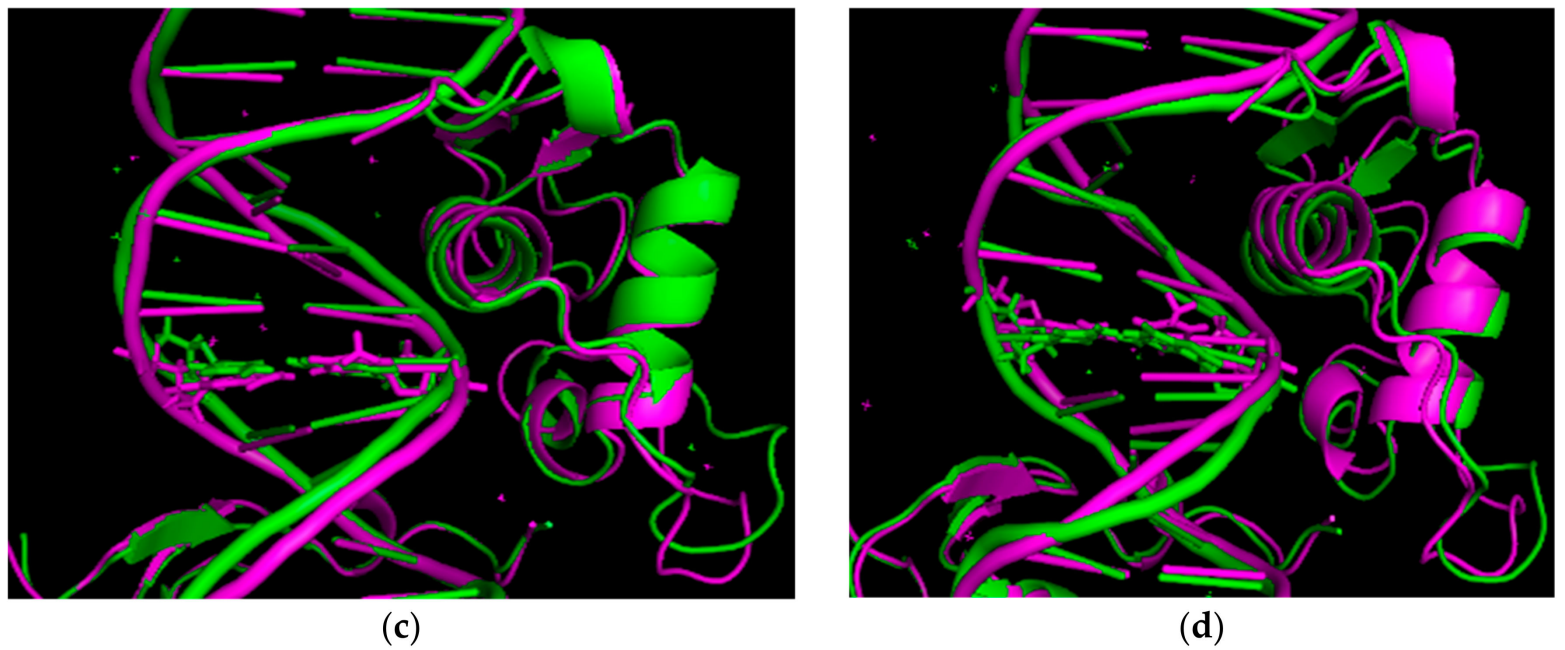
The ultimate frames of the molecular dynamics simulation of HNF-4α DNA complexes: (**a**) Production run 1; (**b**) production run 2; (**c**) production run 3; and (**d**) production run 4. The wild-type complex is depicted in violet, and the complex containing the rSNP is depicted in green. The base pair that gets mutated is shown in sticks, while the remaining base pairs are represented as a ladder.

First, we visually followed the structures of both complexes throughout the molecular dynamics simulation and tried to locate the differences in their conformations. Comparison between the structures of both complexes is shown in Figure 1, where the (mutated) nucleobases represent the center of structures belonging to the ultimate frames of all four molecular dynamics simulation production runs.

Visual inspection of the conformations of both complexes did not reveal any significant differences, as the complexes differ so little and the mutated nucleobases are mostly too far away from the protein part of the complex (the transcription factor HNF-4α) to form any stable interactions.

For the subsequent thermodynamic analysis four systems were needed: Two systems of sole DNA chains (*HNF-1α* gene promotor DNA sequences), one containing the rSNP and the other with the wild-type base pair, and two systems of complexes where the transcription factor HNF-4α was bound to these DNA chains.

Having these four separate systems additionally enabled us to follow the impact of the rSNP on the behavior of sole DNA chains, not only of complexes, which was analyzed through atom-positional root-mean-square deviation (RMSD).

### 3.1. The Results of RMSD Analysis

**Figure 2:**
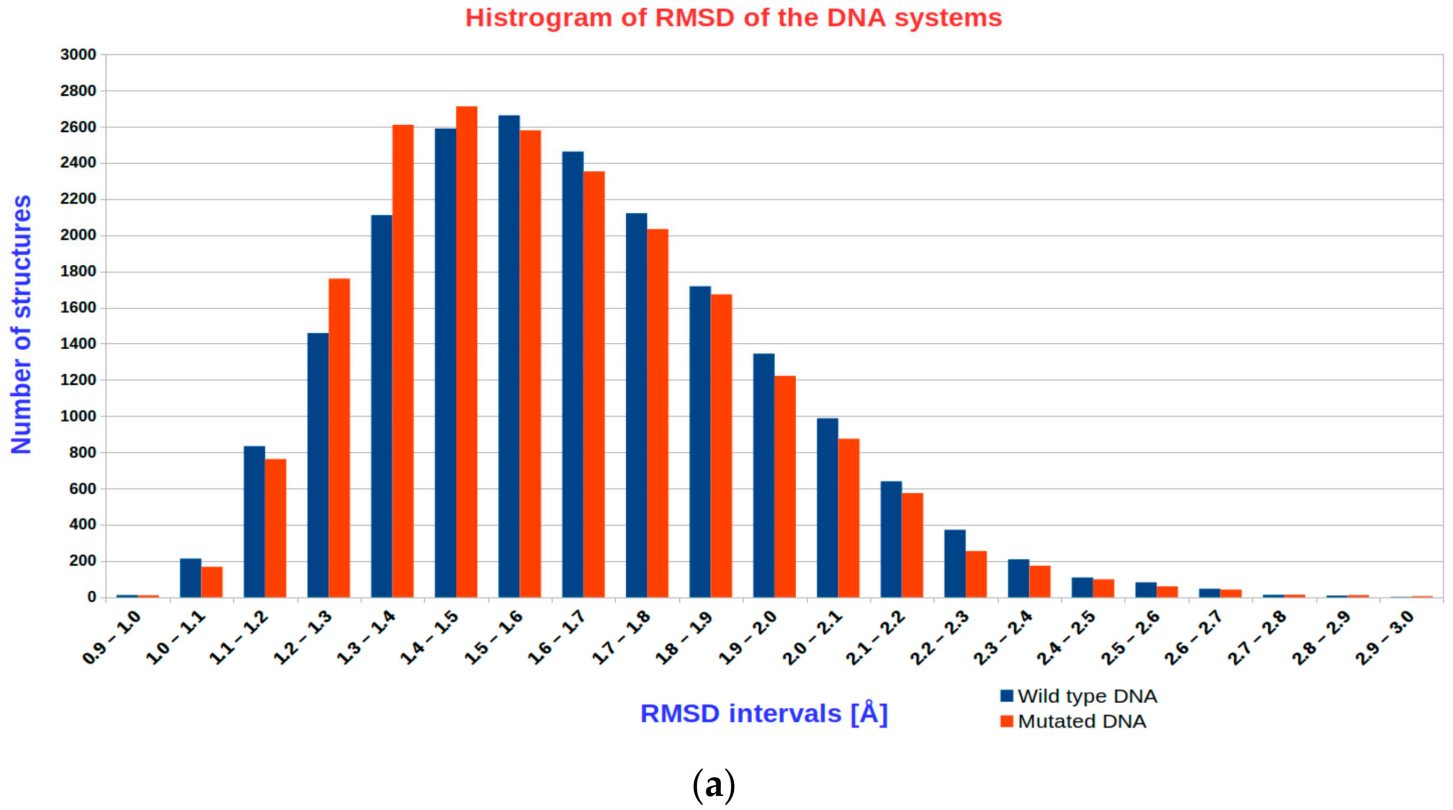

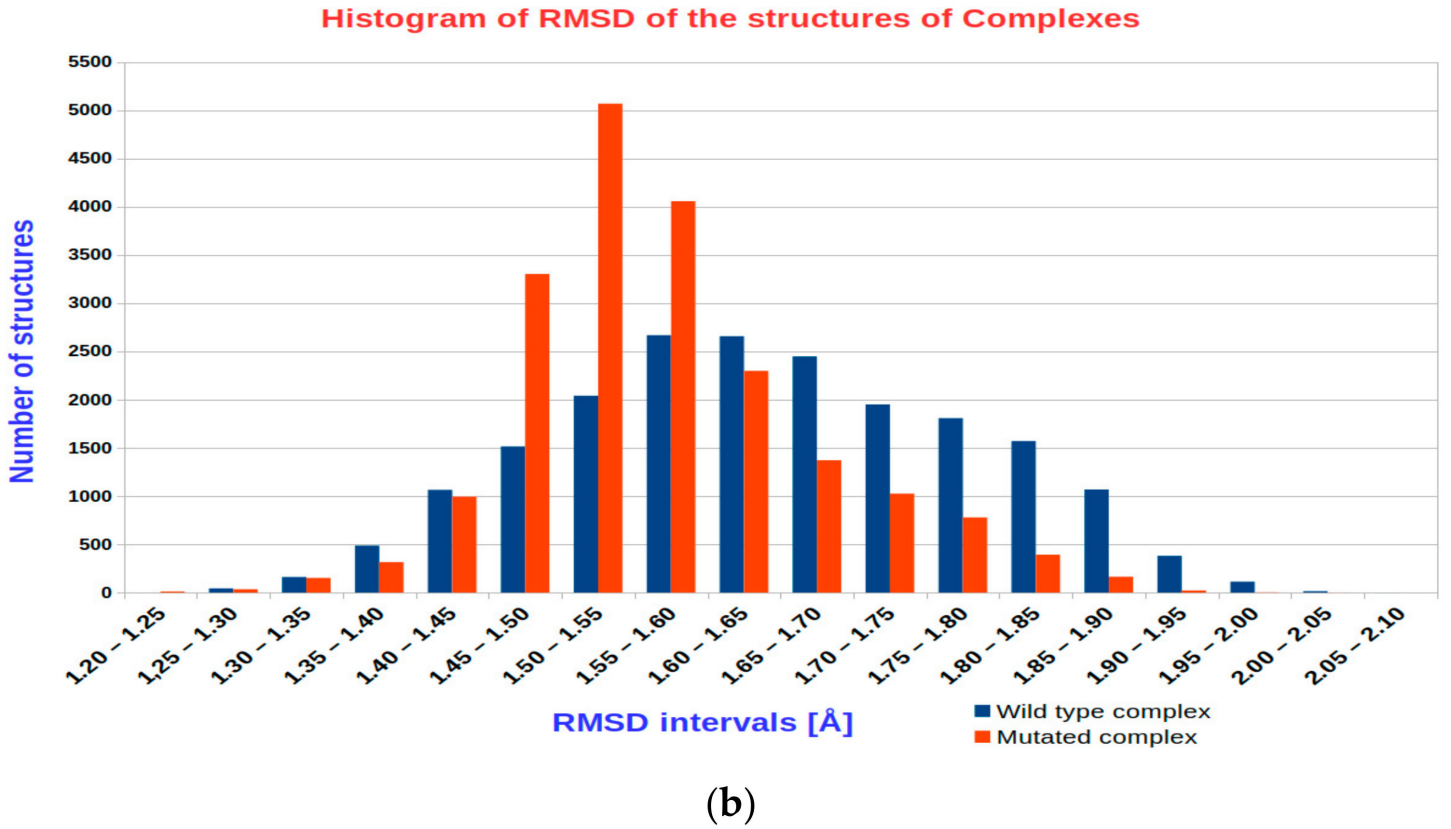
Histograms of root-mean-square difference (RMSD) of atomic positions throughout 16 independent 5 *ns* molecular dynamics simulation production runs of the four investigated systems: (**a**) The wild-type DNA chain containing the *HNF-1α* gene promotor sequence in blue, and the mutated DNA chain containing the *HNF-1α* gene promotor sequence with the rSNP rs35126805 in red color; (**b**) the complex of transcription factor HNF-4α bound to the wild-type DNA *HNF-1α* gene promotor sequence in blue color, and the complex of transcription factor HNF-4α bound to the mutated DNA *HNF-1α* gene promotor sequence with the rSNP rs35126805 in red color.

Figure 2 shows histograms of RMSD of atomic positions throughout 16 independent 5 *ns* molecular dynamics simulation production runs. Under a) the wild-type DNA HNF-1α gene promotor sequence and the DNA containing the rSNP rs35126805 are presented. Under b) wild-type DNA *HNF-1α* gene promotor sequence in complex with transcription factor HNF-4α and its mutated counterpart containing the rSNP rs35126805 are presented.

From Figure 2 and from Supplementary Materials Figure S1, we can observe that in both the mutated DNA containing rSNP rs35126805 and in its complex with transcription factor HNF-4α, the RMSD reaches lower values and fluctuates less, meaning that the structures are more rigid, and consequentially more stable, than their wild-type counterparts. Therefore, the rSNP rs35126805 provides better structural stability to both the DNA and its complex with HNF-4α.

### 3.2. Analysis of Intermolecular Interactions—Hydrogen Bonds

Hydrogen bonds exert a great influence on the binding, especially when dealing with nucleic acids and proteins solvated in water, where they represent vital intra- and intermolecular interactions. Therefore, we examined their quantity, frequency of occurrence, and duration.

At first, the DNA HNF-4α complex with the wild-type base pair adenine–thymine naturally contains fewer hydrogen bonds than the one with mutated rSNP cytosine–guanine base pair as the later forms three hydrogen bonds and the former only two. Moreover, the analysis confirmed that throughout the molecular dynamics simulation the complex with the mutated DNA contains more hydrogen bonds than the wild-type complex thereby explaining its superior structural stability to at least a certain extent. However, as the water molecules in the surrounding solvation sphere are constantly moving and changing their identity, the resulting transient water bridges must exert a certain structure-stabilizing influence as well. [48]

### 3.3. The Results of the Thermodynamical Analysis

The difference in the binding affinity between the wild-type HNF-4α DNA complex and the one with the rSNP rs35126805 mutation was obtained using thermodynamic analysis of the energy trajectory gained during the molecular dynamics simulations of the four investigated systems. As the Linear Interaction Energy (LIE) method was applied the corresponding free energy difference was calculated based on the average electrostatic and van der Waals interaction energies. See Section 2.3.2. of the Methods section and particularly Equations (1) and (2) for more information. In Tables S1–S4 (see Supplementary Materials), the Δ*G_bind_* for all four investigated systems in all the production runs are presented.

In Table 1 the ΔΔ*G_bind_* along with its electrostatic and van der Waals contribution is calculated for all the production runs as a difference between the complex containing rSNP rs35126805 and the complex with the wild-type DNA sequence. However, as pointed out by Smith and van Gunsteren, contributions to free energies are only strictly valid if there is no coupling between them [49].

As we can see from Table 1, the complex containing the rSNP rs35126805 exhibits a lower binding free energy than the wild-type complex, and the difference between them is approximately—0.8 kcal/mol. The negative value means that the complex containing the rSNP is more stable and therefore HNF-4α binds more strongly to the mutated DNA. This difference stems primarily from electrostatic interactions with shape complementarity through van der Waals interactions playing only a minor role. Consequently, the investigated mutation disrupts the metabolic pathway HNF-4α/HNF-1α, causing MODY3 and potentially hepatocellular carcinoma metastasis as well as enhanced apoptosis in pancreatic cancer cells.

The HNF-4α transcription factor forms a homodimer with its plane of symmetry lying one base pair away from the studied rSNP. The studied rSNP rests in the HNF-4α binding site and probably forms the base pair closest to the protein itself, therefore, a measurable effect on the binding affinity can be anticipated. If some other A-T base pair of the HNF-1α regulatory domain would be replaced by a C-G base pair, a smaller binding free energy difference ΔΔ*G_bind_* could be expected.

## 4. Conclusions

To the best of our knowledge, this represents the first study where thermodynamic analyses of the results obtained from molecular dynamics simulations were performed to uncover the influence of rSNP on the protein binding to DNA.

Even though the non-coding part of the genome contains sequences for the regulation of gene expression and according to GWAS germline SNPs located in introns are also associated with altered cancer risks, this research field remains poorly addressed [1,2,3]. Consequentially, only a few available databases on regulatory SNPs (rSNPs) exist. Moreover, structures of complexes between human transcription factors and DNA containing rSNPs are exceedingly rare.

The aim of our study is to elucidate the impact of the MODY3 disease-linked rSNP rs35126805 located in the *HNF-1α* gene promotor on the binding of the transcription factor HNF-4α and consequently on the regulation of *HNF-1α* gene expression. Our focus was to investigate the change in the binding affinity of the transcription factor HNF-4α to the DNA, caused by the rSNP through molecular dynamics simulations and thermodynamic analyses of the acquired results. Both RMSD of atomic positions and the relative difference in the binding free energy ΔΔ*G_bind_* revealed that the HNF-4α binds slightly more strongly to the DNA containing the mutation (rSNP) making the complex more stable/rigid, thereby influencing the expression of the *HNF-1α* gene. This difference mainly originates from electrostatic interactions.

The investigated mutation rSNP rs35126805 disrupts the metabolic pathway HNF-4α/HNF-1α by changing the electrostatic interactions between the HNF-4α transcription factor and the DNA causing MODY3 and potentially also hepatocellular carcinoma metastasis, as well as enhanced apoptosis in pancreatic cancer cells.

As so many questions on the location and biological roles of regulatory SNPs (rSNPs) in disease (cancer) development, progression, and response to therapy remain unanswered, they ought to be investigated in future studies, where novel bsc1 or OL corrections for nucleic acid force fields could be tested out [50,51,52]. Our computational approach was proven generally useful for studying the impact of regulatory single nucleotide polymorphisms on the binding of transcription factors to the DNA.

## Figures and Tables

**Table 1:** The free energy differences ΔΔ*G_bind_* between the complex containing rSNP rs35126805 and the wild-type DNA sequence for all production runs along with their electrostatic and van der Waals components.

Production Run	$\Delta \Delta G_{bind}^{vdW}{\;}^{1}[\mathrm{kcal}/\mathrm{mol}]$	$\Delta \Delta G_{bind}^{es}{\;}^{2}\;[\mathrm{kcal}/\mathrm{mol}]$	$\Delta \Delta G_{bind}$ **[kcal/mol]**
**Production run 1**	−0.20	−0.44	−0.64
**Production run 2**	0.14	−0.92	−0.78
**Production run 3**	−0.31	−0.95	−1.30
**Production run 4**	−0.09	−0.35	−0.44
**Average**	−0.1 ± 0.1	−0.7 ± 0.3	−0.8 ± 0.3

^1^ The van der Waals component of ΔΔ*G_bind_* between the complex containing rSNP rs35126805 and the wild-type DNA sequence. ^2^ The electrostatic component of ΔΔ*G_bind_* between the complex containing rSNP rs35126805 and the wild-type DNA sequence.

## Supplementary Materials

Supplementary materials can be found at https://www.mdpi.com/2218-273X/10/12/1700/s1. Figure S1: RMSD of atomic positions throughout 5 ns molecular dynamics simulation production runs of the four systems, Table S1: The binding free energies of the four studied systems in production run 1 of molecular dynamics simulations, Table S2: The binding free energies of the four studied systems in production run 2 of molecular dynamics simulations, Table S3: The binding free energies of the four studied systems in production run 3 of molecular dynamics simulations, Table S4: The binding free energies of the four studied systems in production run 4 of molecular dynamics simulations.

## Abbreviations

Coot	Crystallographic Object-Oriented Toolkit
Cys	cysteine
GWAS	genome-wide association studies
HNF	hepatocyte nuclear factor
HNF-1α	hepatocyte nuclear factor 1 alpha
HNF-4α	hepatocyte nuclear factor 4 alpha
LIE	Linear Interaction Energy method
MODY3	Maturity-onset diabetes of the young 3
MD	molecular dynamics
PDB	Protein Data Bank
RMSD	root-mean-square deviation
rSNP	regulatory single nucleotide polymorphism
SNP	single nucleotide polymorphism
