# Supplementary material for: Molecular Dynamics Simulations Predict that rSNP Located in the HNF-1α Gene Promotor Region Linked with MODY3 and Hepatocellular Carcinoma Promotes Stronger Binding of the HNF-4α Transcription Factor
Source: Biomolecules. 2020 Dec 21;10(12):1700. doi: 10.3390/biom10121700 (PMC7767403; doi:10.3390/biom10121700)
Supplement: Supplementary file 1 [file biomolecules-10-01700-s001.pdf]

biomolecules

Supplementary materials

Molecular dynamics simulations predict that rSNP located in the *HNF-1α* gene promotor region linked with MODY3 and hepatocellular carcinoma promotes stronger binding of the HNF-4α transcription factor

Eva Španinger [1], Uroš Potočnik [1,2] and Urban Bren [1,3,*]

[1] Faculty of Chemistry and Chemical Engineering, University of Maribor, Smetanova ulica 17, SI-2000 Maribor, Slovenia; eva.spaninger@gmail.com
[2] Faculty of Medicine, University of Maribor, Taborska 8, SI-2000 Maribor, Slovenia; uros.potocnik@um.si
[3] Faculty of Mathematics, Natural Sciences and Information Technologies, University of Primorska, Glagoljaška 8, SI-6000 Koper, Slovenia; urban.bren@um.si
* Correspondence: urban.bren@um.si; Tel.: +386-2-2294-421

Table of Contents

Figure S1. RMSD of atomic positions throughout 5 *ns* molecular dynamics simulation production runs of the four systems 2

Table S1. The binding free energies of the four studied systems in production run 1 of molecular dynamics simulations 3

Table S2. The binding free energies of the four studied systems in production run 2 of molecular dynamics simulations 4

Table S3. The binding free energies of the four studied systems in production run 3 of molecular dynamics simulations 5

Table S4. The binding free energies of the four studied systems in production run 4 of molecular dynamics simulations 6

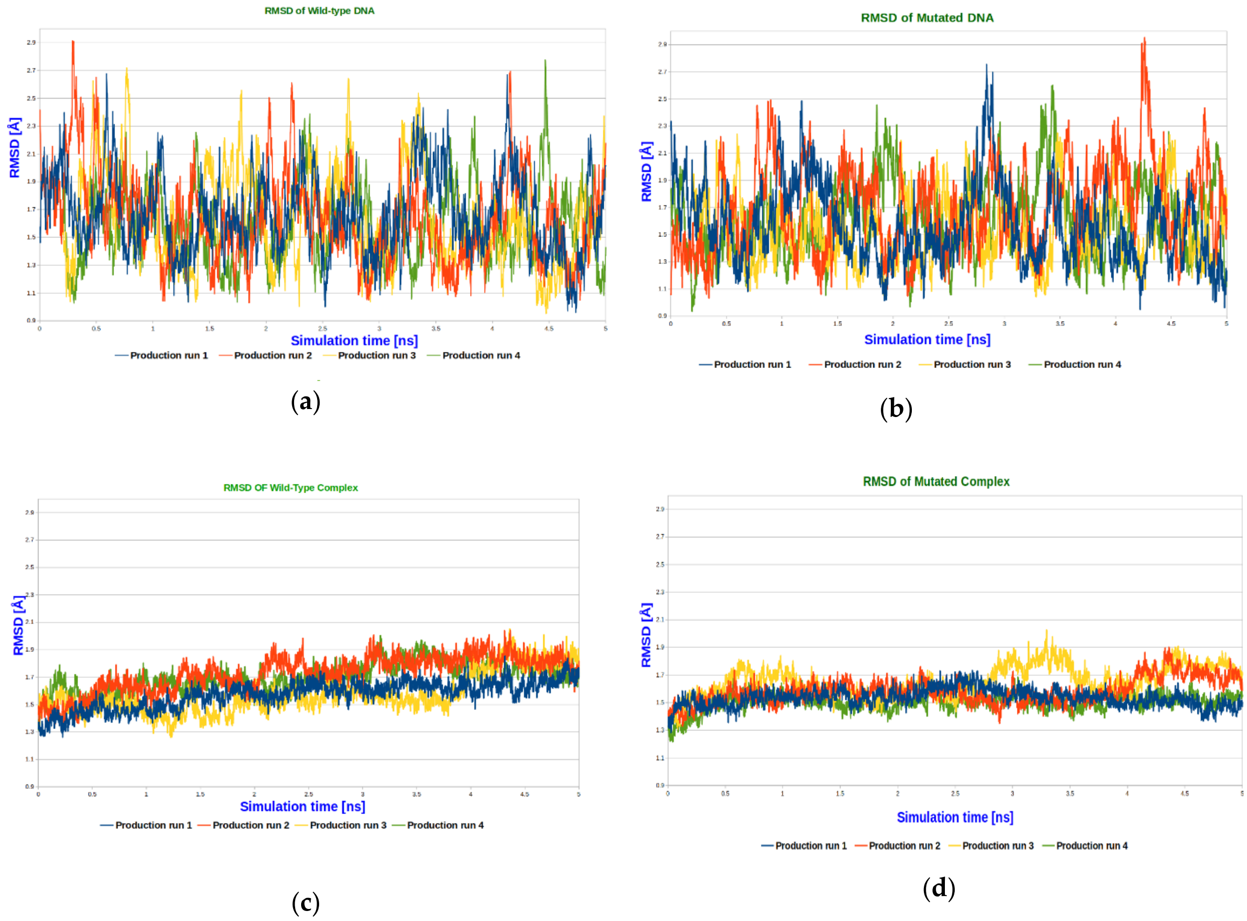

Figure S1. RMSD of atomic positions throughout 5 *ns* molecular dynamics simulation production runs of the four systems: (**a**) The wild-type DNA chain containing the *HNF-1α* gene promotor sequence; (**b**) The mutated DNA chain containing the *HNF-1α* gene promotor sequence with the rSNP rs35126805; (**c**) The complex of transcription factor HNF-4α bound to the wild-type DNA *HNF-1α* gene promotor sequence; (**d**) The complex of transcription factor HNF-4α bound to the mutated DNA *HNF-1α* gene promotor sequence with the rSNP rs35126805. Different colors denote different production runs (1 – green; 2 – orange; 3 - yellow; 4 – blue).

Table S1. The binding free energies of the four studied systems in production run 1 of molecular dynamics simulations.

Simulation time [ns]	ΔG_{bind} [kcal/mol] Mutated complex[1]	Wild-Type complex[2]	Mutated DNA[3]	Wild-Type DNA[4]
0 - 0.2	-28,98	-26,80	-27,83	-28,47
0.2 - 0.4	-28,23	-27,31	-28,04	-28,35
0.4 - 0.6	-28,35	-27,95	-27,60	-29,34
0.6 - 0.8	-28,67	-27,08	-27,91	-29,13
0.8 - 1.0	-28,23	-27,12	-27,86	-29,10
1.0 - 1.2	-27,15	-28,08	-27,99	-29,16
1.2 - 1.4	-27,83	-28,59	-27,76	-28,80
1.4 - 1.6	-27,90	-28,71	-28,41	-29,64
1.6 - 1.8	-28,03	-28,44	-27,84	-29,38
1.8 - 2.0	-28,29	-28,70	-28,16	-29,53
2.0 - 2.2	-27,71	-28,86	-27,95	-29,08
2.2 - 2.4	-28,01	-28,70	-28,11	-29,09
2.4 - 2.6	-27,48	-28,51	-28,41	-29,01
2.6 - 2.8	-27,90	-28,59	-28,13	-28,81
2.8 - 3.0	-28,00	-28,27	-27,33	-28,63
3.0 - 3.2	-28,08	-28,66	-27,45	-28,64
3.2 - 3.4	-27,85	-28,47	-28,16	-28,98
3.4 - 3.6	-27,92	-28,61	-28,05	-29,11
3.6 - 3.8	-28,31	-28,71	-28,25	-29,57
3.8 - 4.0	-27,42	-29,30	-28,04	-29,11
4.0 - 4.2	-27,43	-28,59	-27,74	-28,83
4.2 - 4.4	-27,18	-28,20	-28,31	-29,27
4.4 - 4.6	-27,27	-28,03	-28,25	-28,59
4.6 - 4.8	-26,54	-27,92	-28,05	-29,06
4.8 - 5.0	-27,75	-28,07	-28,29	-29,13
**Averages**				
ΔE_{vdW} [5]	-49,39	-49,59	-46,15	-47,46
ΔE_{el} [6]	-44,11	-44,94	-45,79	-47,65
ΔG_{vdW} [7]	-8,89	-8,93	-8,31	-8,54
ΔG_{el} [8]	-18,97	-19,33	-19,69	-20,49
ΔG_{bind}	**-27,86**	**-28,25**	**-28,00**	**-29,03**
**Standard deviations**				
$s(\Delta E_{vdW})$	0,66	0,33	0,63	0,65
$s(\Delta E_{el})$	1,12	1,41	0,81	0,79
$s(\Delta G_{vdW})$	0,12	0,06	0,11	0,12
$s(\Delta G_{el})$	0,48	0,60	0,35	0,34
$s(\Delta G_{bind})$	**0,52**	**0,61**	**0,28**	**0,33**

[1] DNA-HNF-4α complex containing rSNP rs35126805. [2] DNA-HNF-4α complex with the wild-type base pair. [3] HNF-1α gene promotor DNA sequence containing rSNP rs35126805. [4] HNF-1α gene promotor DNA sequence. [5] Average van der Waals interaction energies between the ligand and its surrounding (kcal/mol). [6] Average electrostatic interaction energies between the ligand and its surrounding (kcal/mol). [7] The van der Waals component of the binding free energy ΔG_{bind} (kcal/mol). [8] The electrostatic component of the binding free energy ΔG_{bind} (kcal/mol).

Table S2. The binding free energies of the four studied systems in production run 2 of molecular dynamics simulations.

Simulation time [ns]	ΔG_{bind} [kcal/mol]			
	**Mutated complex**[1]	**Wild-Type complex**[2]	**Mutated DNA**[3]	**Wild-Type DNA**[4]
0 - 0.2	-27,14	-28,05	-27,91	-29,64
0.2 - 0.4	-27,45	-27,39	-27,89	-28,65
0.4 - 0.6	-27,41	-27,04	-27,80	-28,74
0.6 - 0.8	-27,50	-27,49	-27,97	-29,29
0.8 - 1.0	-27,30	-27,80	-27,64	-29,10
1.0 - 1.2	-28,42	-28,00	-28,00	-29,37
1.2 - 1.4	-27,74	-28,74	-28,04	-29,01
1.4 - 1.6	-27,25	-28,47	-27,99	-29,07
1.6 - 1.8	-28,38	-28,14	-28,04	-28,77
1.8 - 2.0	-27,45	-27,81	-28,11	-29,30
2.0 - 2.2	-27,48	-27,56	-27,41	-29,76
2.2 - 2.4	-27,70	-27,86	-28,18	-29,57
2.4 - 2.6	-26,37	-28,22	-27,80	-29,52
2.6 - 2.8	-27,55	-29,35	-28,28	-29,64
2.8 - 3.0	-29,12	-28,32	-27,95	-29,49
3.0 - 3.2	-27,14	-28,24	-27,20	-29,40
3.2 - 3.4	-27,07	-28,58	-27,66	-28,86
3.4 - 3.6	-26,76	-27,85	-27,74	-28,72
3.6 - 3.8	-27,20	-28,37	-28,01	-29,33
3.8 - 4.0	-26,79	-28,76	-27,29	-29,13
4.0 - 4.2	-28,32	-28,76	-27,09	-29,89
4.2 - 4.4	-26,95	-28,93	-27,44	-30,62
4.4 - 4.6	-27,84	-28,55	-26,62	-29,48
4.6 - 4.8	-27,11	-28,7966	-27,61	-29,32
4.8 - 5.0	-26,97	-29,0276	-27,44	-28,72
**Averages**				
ΔE_{vdW}[5]	-47,84	-49,78	-46,46	-47,62
ΔE_{el}[6]	-43,83	-44,85	-45,03	-48,19
ΔG_{vdW}[7]	-8,61	-8,96	-8,36	-8,57
ΔG_{el}[8]	-18,85	-19,28	-19,36	-20,72
ΔG_{bind}	**-27,46**	**-28,24**	**-27,73**	**-29,30**
**Standard deviations**				
$s(\Delta E_{vdW})$	0,47	0,52	0,51	0,61
$s(\Delta E_{el})$	1,48	1,28	1,05	0,93
$s(\Delta G_{vdW})$	0,08	0,09	0,09	0,11
$s(\Delta G_{el})$	0,63	0,55	0,45	0,40
$s(\Delta G_{bind})$	**0,61**	**0,57**	**0,39**	**0,45**

[1] DNA-HNF-4α complex containing rSNP rs35126805. [2] DNA-HNF-4α complex with the wild-type base pair. [3] HNF-1α gene promotor DNA sequence containing rSNP rs35126805. [4] HNF-1α gene promotor DNA sequence. [5] Average van der Waals interaction energies between the ligand and its surrounding (kcal/mol). [6] Average electrostatic interaction energies between the ligand and its surrounding (kcal/mol). [7] The van der Waals component of the binding free energy ΔG_{bind} (kcal/mol). [8] The electrostatic component of the binding free energy ΔG_{bind} (kcal/mol).

Table S3. The binding free energies of the four studied systems in production run 3 of molecular dynamics simulations.

Simulation time [ns]	ΔG_{bind} [kcal/mol]			
	**Mutated complex**[1]	**Wild-Type complex**[2]	**Mutated DNA**[3]	**Wild-Type DNA**[4]
0 - 0.2	-27,69	-28,23	-28,27	-28,79
0.2 - 0.4	-28,30	-28,80	-26,91	-28,76
0.4 - 0.6	-28,58	-28,28	-27,84	-29,38
0.6 - 0.8	-28,50	-28,41	-28,53	-29,22
0.8 - 1.0	-28,30	-28,87	-28,33	-29,11
1.0 - 1.2	-28,29	-28,59	-28,01	-29,06
1.2 - 1.4	-27,59	-29,06	-28,50	-29,19
1.4 - 1.6	-28,28	-28,55	-28,16	-29,16
1.6 - 1.8	-28,36	-29,02	-28,31	-28,88
1.8 - 2.0	-28,40	-28,75	-28,26	-29,82
2.0 - 2.2	-28,36	-28,92	-28,00	-29,27
2.2 - 2.4	-28,24	-28,86	-28,02	-29,90
2.4 - 2.6	-28,13	-28,81	-28,28	-29,71
2.6 - 2.8	-27,64	-28,13	-27,95	-30,47
2.8 - 3.0	-27,69	-28,63	-28,14	-30,50
3.0 - 3.2	-28,01	-28,78	-27,88	-30,07
3.2 - 3.4	-28,52	-28,50	-28,06	-29,01
3.4 - 3.6	-27,86	-28,63	-28,23	-29,89
3.6 - 3.8	-27,62	-28,04	-28,31	-31,17
3.8 - 4.0	-27,21	-28,22	-28,48	-31,08
4.0 - 4.2	-28,11	-28,27	-27,99	-30,59
4.2 - 4.4	-28,33	-27,87	-28,51	-30,91
4.4 - 4.6	-28,54	-28,03	-27,66	-30,61
4.6 - 4.8	-29,21	-29,01	-27,94	-29,52
4.8 - 5.0	-28,81	-28,09	-27,82	-28,55
**Averages**				
ΔE_{vdW}[5]	-49,79	-49,72	-46,20	-47,87
ΔE_{el}[6]	-44,70	-45,54	-46,00	-49,04
ΔG_{vdW}[7]	-8,96	-8,95	-8,32	-8,62
ΔG_{el}[8]	-19,22	-19,58	-19,78	-21,09
ΔG_{bind}	**-28,18**	**-28,53**	**-28,10**	**-29,71**
**Standard deviations**				
$s(\Delta E_{vdW})$	0,58	0,60	0,66	0,78
$s(\Delta E_{el})$	1,11	0,90	0,79	1,91
$s(\Delta G_{vdW})$	0,10	0,11	0,12	0,14
$s(\Delta G_{el})$	0,48	0,39	0,34	0,82
$s(\Delta G_{bind})$	**0,44**	**0,35**	**0,34**	**0,78**

[1] DNA-HNF-4α complex containing rSNP rs35126805. [2] DNA-HNF-4α complex with the wild-type base pair. [3] HNF-1α gene promotor DNA sequence containing rSNP rs35126805. [4] HNF-1α gene promotor DNA sequence. [5] Average van der Waals interaction energies between the ligand and its surrounding (kcal/mol). [6] Average electrostatic interaction energies between the ligand and its surrounding (kcal/mol). [7] The van der Waals component of the binding free energy ΔG_{bind} (kcal/mol). [8] The electrostatic component of the binding free energy ΔG_{bind} (kcal/mol).

Table S4. The binding free energies of the four studied systems in production run 4 of molecular dynamics simulations.

Simulation time [ns]	ΔG_{bind} [kcal/mol]			
	**Mutated complex**[1]	**Wild-Type complex**[2]	**Mutated DNA**[3]	**Wild-Type DNA**[4]
0 - 0.2	-27,84	-28,78	-27,53	-29,60
0.2 - 0.4	-27,98	-29,07	-27,89	-28,99
0.4 - 0.6	-28,23	-28,65	-27,93	-29,34
0.6 - 0.8	-27,46	-28,43	-28,34	-29,61
0.8 - 1.0	-27,83	-29,11	-27,86	-29,02
1.0 - 1.2	-27,07	-28,61	-27,35	-28,92
1.2 - 1.4	-27,71	-28,72	-27,21	-29,48
1.4 - 1.6	-27,81	-28,64	-28,30	-30,07
1.6 - 1.8	-28,84	-28,78	-28,05	-29,87
1.8 - 2.0	-29,04	-28,50	-29,49	-29,57
2.0 - 2.2	-28,31	-28,69	-30,75	-29,90
2.2 - 2.4	-27,74	-29,24	-29,15	-30,47
2.4 - 2.6	-26,61	-29,33	-28,24	-30,23
2.6 - 2.8	-27,15	-29,75	-28,25	-29,68
2.8 - 3.0	-27,27	-28,58	-28,12	-29,46
3.0 - 3.2	-27,43	-29,18	-27,83	-30,18
3.2 - 3.4	-28,93	-29,34	-27,51	-29,69
3.4 - 3.6	-29,16	-28,88	-28,36	-29,64
3.6 - 3.8	-28,39	-29,15	-27,89	-29,11
3.8 - 4.0	-28,65	-29,47	-28,05	-28,77
4.0 - 4.2	-28,38	-29,99	-27,94	-28,76
4.2 - 4.4	-28,16	-29,09	-27,66	-29,71
4.4 - 4.6	-27,22	-28,98	-27,48	-29,44
4.6 - 4.8	-27,13	-28,53	-27,91	-30,26
4.8 - 5.0	-26,71	-28,43	-28,03	-31,07
**Averages**				
ΔE_{vdW}[5]	-49,02	-49,59	-47,22	-48,28
ΔE_{el}[6]	-44,33	-46,58	-45,64	-48,70
ΔG_{vdW}[7]	-8,82	-8,93	-8,50	-8,69
ΔG_{el}[8]	-19,06	-20,03	-19,63	-20,94
ΔG_{bind}	**-27,88**	**-28,96**	**-28,13**	**-29,63**
**Standard deviations**				
$s(\Delta E_{vdW})$	0,59	0,48	0,98	0,52
$s(\Delta E_{el})$	1,64	1,09	1,93	1,20
$s(\Delta G_{vdW})$	0,11	0,09	0,18	0,09
$s(\Delta G_{el})$	0,71	0,47	0,83	0,51
$s(\Delta G_{bind})$	**0,72**	**0,41**	**0,74**	**0,56**

[1] DNA-HNF-4α complex containing rSNP rs35126805. [2] DNA-HNF-4α complex with the wild-type base pair. [3] HNF-1α gene promotor DNA sequence containing rSNP rs35126805. [4] HNF-1α gene promotor DNA sequence. [5] Average van der Waals interaction energies between the ligand and its surrounding (kcal/mol). [6] Average electrostatic interaction energies between the ligand and its surrounding (kcal/mol). [7] The van der Waals component of the binding free energy ΔG_{bind} (kcal/mol). [8] The electrostatic component of the binding free energy ΔG_{bind} (kcal/mol).

Publisher's Note: MDPI stays neutral with regard to jurisdictional claims in published maps and institutional affiliations.

© 2020 by the authors. Licensee MDPI, Basel, Switzerland. This article is an open access article distributed under the terms and conditions of the Creative Commons Attribution (CC BY) license (http://creativecommons.org/licenses/by/4.0/).
